# The Ideological Turing Test: A Behavioral Measure of Open‐Mindedness and Perspective‐Taking

**DOI:** 10.1111/cogs.70126

**Published:** 2025-10-14

**Authors:** Charlotte O. Brand, Daniel Brady, Tom Stafford

**Affiliations:** ^1^ School of Psychology The University of Sheffield; ^2^ Research Software Engineering & School of Computer Science The University of Sheffield

**Keywords:** Reasoning, Argumentation, Dialog, Open‐mindedness, Perspective‐taking, Actively open‐minded thinking, Polarization

## Abstract

Understanding our ideological opponents is crucial for the effective exchange of arguments and the avoidance of escalation, and the reduction of conflict. We operationalize the idea of an “Ideological Turing Test” to measure the accuracy with which people represent the arguments of their ideological opponents. Crucially, this offers a behavioral measure of open‐mindedness which goes beyond mere self‐report. We recruited 200 participants from opposite sides of three topics with potential for polarization in the UK of the early 2020s (1200 participants total). Participants were asked to provide reasons both for and against their position. Their reasons were then rated by participants from the opposite side. Our criteria for “passing” the test was if an argument was agreed with by opponents to the same extent or higher than arguments made by proponents. We found evidence for high levels of mutual understanding across all three topics. We also found that those who passed were more open‐minded toward their opponents, in that they were less likely to rate them as ignorant, immoral, or irrational. Our method provides a behavioral measure of open‐mindedness and ability to mimic counterpartisan perspectives that goes beyond self‐report measures. Our results offer encouragement that, even in highly polarized debates, high levels of mutual understanding persist.

## 1 Introduction

The exchange of reasons is recognized as central to our intellectual and political lives (Mercier & Sperber, 2017; Habermas, 1991; Singer, 1981). Reasoning can have a coordinating function, but an excess of this coordination can drive polarization, as social pressure encourages conformity, including in the expression of reasons (Noelle‐Neumann, 1974). Once polarized groups have formed, individuals may share reasons for identity, expressive rather than truth‐seeking purposes (Kahan, 2015), so‐called expressive responding (Schaffner & Luks, 2018).

The wider function of collective intelligence can be fueled by dissenting views, and even polarization, among individuals (Galton, 1907; Surowiecki, 2004). This is especially so when disagreement is harnessed by deliberation (Landemore, 2012), but this assumes exchange and comprehension of reasons across lines of disagreement. The social fact of ideological and affective polarization may hinder that comprehension, a possibility which seems exacerbated by the contemporary state of (social) media (but see Boxell, Gentzkow, & Shapiro, 2024).

Thus, many prominent topics in public debate can seem polarized such that the proponents and opponents are assumed to disagree vehemently with little to no middle ground being acknowledged or expressed (Ross Arguedas, Robertson, Fletcher, & Nielsen, 2022). Any tendency to overestimate the distance between opposing views can lead to complementary and misrepresentative stereotypes on both sides of a debate (e.g., Mintchev, 2021; Wiwad, Waldfogel, Shariff, & Kteily, 2025). Reducing misunderstandings, identifying areas of agreement, and preventing polarization relies on an accurate representation of what your ideological opponents truly think. Although discussion and debate requires the exchange of arguments and counterarguments (Mercier, 2016), these counterarguments need to first be grounded in an accurate representation of the opponent's views. Failure to do this represents not only a missed opportunity to engage, it can drive polarization through identity‐expressive (Kahan, 2015) rather than truth seeking communications, as well as contribute to pluralistic ignorance (O'Gorman, 1986), whereby most people fail to recognize the true beliefs of others (Levendusky & Malhotra, 2016; Mildenberger & Tingley, 2019).

Whether we truly understand those we disagree with well enough to engage in successful debate is rarely questioned before entering into a debate to begin with, often leading to escalation. The current paper explores whether it is possible to successfully measure how well individuals understand the reasons of their ideological opponents.

### 1.1 The need for an Ideological Turing Test

One of the rules put forward by Daniel Dennett for anyone about to engage in debate is “You should attempt to re‐express your target's position so clearly, vividly, and fairly that your target says, ‘Thanks, I wish I'd thought of putting it that way’,” (Dennett, 2013). The idea is echoed in the strategy, popular among so‐called “rationalist” communities, of constructing a “steel man” version of a position—the strongest possible characterization of an argument (and the opposite of the “straw man” position, where a misrepresentation is used to fallaciously score debating points (Lewiński, 2011). Guidance for Wikipedia editors (see De Kock, Stafford, & Vlachos, 2022) suggests that productive disagreement should occupy the top levels of a hierarchy of disagreement (Graham, 2008), aspiring to “refute the central point,” but to achieve this, you must first have an accurate and fair representation of the opponent's central point. Sentiments such as these have fed into the suggestion of an “Ideological Turing Test” (ITT)—a play on the classic “Turing Test” (Turing, 1950) in which an artificial intelligence must convince a human that they are human. The ITT, then, entails convincing an ideological opponent that you share their beliefs (Caplan, 2011, Econlib).

To our knowledge, no one has operationalized the ITT to allow formal assessment of how well an individual, or a group, can pass this test. Nor has it been established how this measure tracks related self‐report measures such as “open‐mindedness” (e.g., Baron, 1993; Stanovich & West 1997, see review below). A behavioral measure would not only allow an assessment of the baseline ability to pass this test by different cohorts, but would also allow researchers to more directly measure the success of interventions which propose to increase a person or a group's ability to take another's perspective; something which is argued to be a fundamental component of “open‐mindedness” or “rational thinking.” Problems with self‐report measures have been known to researchers for many decades (Walsh, 1967; Baker & Brandon, 1990; Peterson & Kerin, 1981). Some prominent issues include demand effects—participants behaving how they assume the researcher wants them to behave (Corneille &Lush, 2023), social desirability bias—participants want to appear more in line with the desirable social norm than they actually are (Paulhus, 2001), and response shift bias—participants do not answer consistently when asked at a different time/in a different context (Howard & Dailey 1979). Evidence shows that participants may have limited insight into their reported attitudes, and those attitudes consequently have limited stability (Hall, Johansson, & Strandberg, 2012). Accordingly, papers show self‐report scales to be inaccurate in a variety of domains; from depression and mental health (Taylor & Brown 1988), environmental behavior (Kormos & Gifford 2014), to health behavior (Del Boca & Noll 2000), and management and leadership studies (Peterson & Kerin 1981). We suggest that a self‐report scale is an inappropriate measure for an ability which relies on one's capacity to “step outside of yourself,” as this metacognitive introspection is inherently what is being measured with any self‐report scale. In contrast to self‐report measures, a strong behavioral measure could only be improved by a genuine increase in the underlying ability to understand and represent contrary points of view, and would not be as susceptible to failures in metacognition or introspection.

### 1.2 Measuring open‐mindedness

Open‐mindedness is typically viewed as a virtuous quality to have, in that it is typically associated with a person's ability to be nonjudgmental about those who hold another point of view to their own, to listen to new evidence that is counter to their current belief, and to be willing to change their mind based on this evidence. One of the most pervasive measures related to the latter of these facets of open‐mindedness in psychology is the “actively open‐mindedness” (AOT) scale. The term was first coined by Baron (1993) and focused on the ability to weigh new evidence heavily against one's prior beliefs. To try and capture individual differences in this type of critical thinking, a 56‐item self‐report scale was developed by Stanovich and West (1997), and refined to 47 items by the same authors in 2008. The scale involves items such as “People should always take into consideration evidence that goes against their beliefs,” and “Changing your mind is a sign of weakness” (the latter reverse scored). The AOT has been found to correlate with a range of tasks, such as evaluating argument quality independently to whether they align with one's own view (Stanovich & West, 1997; Stanovich & West, 2008), many types of critical thinking tasks (Campitelli & Gerrans, 2014; Heijltjes et al, 2014; Macpherson & Stanovich, 2007; Sá & Stanovich, Sinatra, Southerland, McConaughy, & Demastes, 2003; West, Toplak, & Stanovich, 2008), and avoidance of conspiratorial or superstitious thinking (Stanovich, West, & Toplak, 2016; Svedholm & Lindeman 2013; Swami, Voracek, Stieger, Tran, & Furnham, 2014).

A recent attempt to refine the 47‐item scale further by Svedholm‐Häkkinen and Lindeman (2018) found that the AOT is not a unitary measure but consists of four distinct dimensions; these dimensions relate to attitudes toward other people as well as attitudes toward knowledge: dogmatism (a lack of dogmatic thinking), fact resistance (an openness to change one's mind in the face of facts), liberalism (liberal and tolerant attitudes toward other people), and belief personification (a refusal to judge people for their opinions). Svedholm‐Häkkinen and Lindeman (2018) used a total sample of 3345 participants from both student, nonstudent, adult, and adolescent populations to confirm their factor analysis, and found internal validity scores for each subscale as great as the original AOT scales. The authors argue that the dimensionality of the original AOT scale had never been thoroughly reported and that the original scale was composed of many different sources.

### 1.3 Reassessments of the AOT scale

In the same vein as Svedholm‐Håkkinen and Lindemen's analysis above, Janssen et al. (2020) thoroughly investigated the psychometric properties of the AOT scale and could not provide support for the hypothesis that either the 41‐item AOT or a subset of items could measure “actively open‐minded thinking” as a single latent trait. They stated that “*there is no item set of the 41 item version of the AOT that can be used to validly order individuals on their ability to think actively open‐mindedly…Consequently, it is questionable whether scores on the AOT provide insights into the concept it aims to measure. If the results of the present analyses replicate … this would be a strong argument for starting the process of (re‐)designing a new scale to measure actively open‐minded thinking or to consider alternative measures*.” To arrive at this conclusion, the authors conducted a Mokken scale analysis; in contrast to previously used Factor Analysis, this analysis does not require multivariate normality or linear correlation between items, which are two assumptions that are violated when working with Likert scale data. Regarding the social and interrelational side of the AOT scale, some scholars have recently attempted to develop scales of “intellectual humility (IH),” in which the focus is on recognizing that your own views may be wrong, and respecting the views of others. Krumrei‐Mancuso and Rouse (2016) developed and validated a 22‐item IH scale, in which they compare this scale to the original AOT scale as part of their validation. However, this study was included in the aforementioned Jenssen et al. review, suggesting it may also have validity issues in that it used inappropriate Factor Analyses for Likert‐scale data. Leary et al. (2017) developed their own 6‐item IH scale (reportedly simultaneously to Krumrei‐Mancuso & Rouse, as mentioned in their acknowledgments), which they report has an “exceptional alpha for such a short scale,” however, curiously, this study does not acknowledge or reference the AOT scales mentioned above, despite obvious overlap.

Independently of issues with scale validity or reliability, both AOT and IH scales are self‐report, and so vulnerable to the limitations of self‐report measures identified in the short review above. Arguably, since both concern normative topics—who would wish to self‐report that they are intellectually arrogant or close‐minded?—Both may be at excess risk of demand effects.

### 1.4 Developing a behavioral measure of open‐mindedness

Instead of relying on self‐report scales with their aforementioned drawbacks, in this study, we endeavored to develop a *behavioral measure* of open‐mindedness, namely, the Ideological Turing Test (hereafter ITT). In brief, we operationalize passing the ITT as the ability to produce arguments that your ideological opponents agree with at the same rate, or higher, as they agree with arguments made by people who genuinely hold that view.

Given the above criticisms of current AOT scales, and to bring the focus of open‐mindedness back to the ability to understand another's perspective (rather than introspective reflections), we test our behavioral measure against a recently developed measure of attitudes toward ideological opponents (Stanley, Whitehead, Sinnott‐Armstrong, & Seli, 2020). In Stanley et al.’s measure, participants are asked about their attitudes toward their ideological opponents, for example, vegans are asked to what extent they agree that non‐vegans are ignorant, irrational, immoral, and so on. Stanley's measure was also tested alongside exposure to ideological opponent's reasons; thus, participants were asked to what extent they agree that their ideological opponents have good reasons for their position. We choose these measures to test alongside our ITT as a more precise probe into one's ability to be open‐minded toward ideological opponents, specifically.

Participants can pass or fail the ITT based on their ability to literally mimic the perspective of the other side. While any test of mimicry must remain silent on the ultimate true belief of the mimic, we note that perspective‐taking is fundamental to human coordination and communication (Tomasello, 2010), and, as such, the ability to accurately represent (mimic) others’ beliefs is necessary if not sufficient for productive disagreement.

We recruit participants from opposite sides of typically polarizing topics to provide reasons both for their own position (proponent reasons), and reasons for the opposite position (opponent reasons). These arguments are then rated by those who are both on their side (proponent) and the opposite side (opponent) of a debate. We operationalize “passing” the ITT as the ability to provide an opponents’ reason that is agreed with to the same (or higher) extent as a reason provided by a genuine opponent, rated by opponents. We also use an “absolute” passing criteria that is not tied to proponent ratings, detailed below. We hypothesize that the ability to “pass” the ITT test varies according to an individual's engagement with the topic (e.g., reading blogposts, listening to podcasts), as well as their experience in discussing the topic with those who disagree with them. We also hypothesize that passing the ITT test correlates positively with a person's judgments of their ideological opponents (Stanley et al., 2020). Throughout the study, we select our wording carefully to represent participants’ perspective‐taking ability, rather than their reasoning ability. As such, participants are asked to “*provide three reasons person X might give*” rather than “*provide three good reasons for position X*,” and likewise, raters are asked how strongly *they agree* with reason X, not whether they think X is a good/valid/true reason.

## 2 Method

All hypotheses, predictions, analysis plans, and sample size rationale were preregistered and are openly available on the Open Science Framework here: https://osf.io/uz9ge

All data and analysis scripts are available at https://doi.org/10.5281/zenodo.16872578. A shinyapp which allows interactive exploration of the arguments participants provided, and their ratings, is available here: https://sheffield‐university.shinyapps.io/OuMshiny/

For clarity, we refer to the position of a written argument as “Argument Position,” the position of the person writing the argument as “Arguer Position,” and the position of the person rating the argument as “Rater Position.” Those providing arguments consistent with their own position are termed “proponents,” and those who are in opposition to that argument are termed “opponents.” Thus, arguments may be “Proponent arguments” (arguer‐argument congruent) or “Opponent arguments” (arguer‐argument incongruent).

### 2.1 Design

#### 2.1.1 Collecting arguments

Six hundred participants were recruited via Prolific and prescreened to be either Vegan (100) or Non‐Vegan (100), For the Covid‐19 vaccines (100) or Against Covid‐19 vaccines (100), and a Leaver in the Brexit vote (100), or a Remainer in the Brexit vote (100). Each participant was asked about their position at the beginning of the study to confirm prescreening consistency within Prolific profiles, and also asked to provide how confident they are in their position on a 7‐point Likert scale. Participants were asked to provide three separate arguments for their own position (henceforth referred to as Proponent Argument), and then to provide three separate arguments for the opposite position (henceforth referred to as Opponent Argument). Specifically, they were asked to imagine talking with someone on each side of the debate, and asked to imagine what reasons that person might give for their position. For example, in the Vegan topic, vegan participants were first asked:

*“Imagine you are chatting with someone who is vegan. What might this person say to you? Please provide THREE reasons that they might give for being vegan in the boxes below.”*

And then on a subsequent page, asked:

*“Imagine you are chatting with someone who is NOT vegan. What might this person say to you? Please provide THREE reasons that they might give for NOT being vegan in the boxes below.”*

Participants were then required to rate how often they actively research the topic (e.g., read books/watch documentaries/listen to podcasts/read articles) and how often they discuss the topic with those who disagree with them, on a 7‐point Likert scale: (1 = never, 2 = rarely, 3 = once or twice a year, 4 = a few times a year, 5 = most months, 6 = most weeks 7 = most days). Participants were then given the opportunity (but not required) to provide their age, gender, and educational level.

Before providing their arguments, participants in the Vegan and Brexit topics were also asked to rate their “ideological opponents” (those who are on the opposite side of the debate) according to measures used by Stanley et al. (2020) (e.g., “To what extent do you agree that vegans have good reasons for their position?” and “To what extent do you agree that vegans are [ignorant/unintelligent/irrational and immoral/unethical/of bad moral character],” all rated on a 7‐point Likert scale from Strongly Disagree to Strongly Agree). Arguments in the Covid‐19 Vaccine topic were collected as part of a previous, unrelated study (Brand & Stafford, 2022), which did not include these Stanley measures.

Two attention check questions were randomly assigned throughout the task that stated “We would like to check you are reading each question carefully, please select ‘Somewhat Agree’ for this answer” and the second requesting “Somewhat Disagree” be selected. These questions mimicked the format and layout of all other Likert scale response questions, so as not to be conspicuous.

We excluded participants who failed both of our attention checks and/or who provided nonsensical answers in the free response questions, as stated in the preregistration.

#### 2.1.2 Rating arguments

Twelve hundred different participants were recruited via Prolific to be either Vegan (200), Non‐vegan (200), For the Covid‐19 vaccines (200) or Against the Covid‐19 vaccines (200), a Remainer in the Brexit vote (200), or a Leaver in the Brexit vote (200). Participants were also prescreened to ensure they were not part of any of the studies that collected the arguments beforehand (thus, participants would not be rating their own arguments at any point).

The 1200 arguments for each topic were randomized into batches of 60 arguments, so that each argument was rated by 10 different participants from each side of the debate, and that each rater rated 60 different arguments each. For example, each pro‐vegan argument written by a vegan was rated by 10 different vegans and 10 different non‐vegans, so that every argument was independently rated 20 times in total.

Crucially, participants were *not* told whether the arguments they were rating were written by people who were for or against a particular topic. Their instructions were as follows:

*“On the following pages you will see arguments for or against being vegan. These reasons have been written previously by other people and have not been fact‐checked. Your task is to say how strongly you agree or disagree with these statements as reasons for or against being vegan.”*

Importantly, these instructions require participants to state how strongly *they* agree or disagree with the reason, not how “good,” “valid,” or “true” these reasons are. This is to aptly reflect the argument collection phase of the study, in which participants are asked to “*imagine you are talking to X, provide three reasons X might give*.” Participants rated their agreement on a 7‐point Likert scale (Strongly Disagree, Disagree, Somewhat Disagree, Neither Agree nor Disagree, Somewhat Agree, Agree, and Strongly Agree).

The arguments were proofread and processed beforehand to check that participants had not written in the third person rather than the first person. We deliberately phrased our question to encourage reason‐givers to write the reasons in the first person; however, this was not always the case. To ensure the raters were not aware of the position of the writer, and that arguments appeared to be genuinely expressed, all arguments were converted to the first person. For example, “they might be scared of needles” was changed to “I am scared of needles.” Many arguments contained sarcastic or derogatory comments toward their opponents alongside the content of the argument, which was removed to avoid biasing the rating, for example, “They are one of those idiots who thinks they will become magnetic or have trackers in them” was changed to “I think I will become magnetic or have trackers in me.”

We excluded participants who failed both of our attention checks (two questions embedded throughout the ratings as before) and/or who provided inconsistent ratings (e.g., a single participant rating “vaccines aren't safe” as both “agree” and “disagree” within the same batch). We manually checked all participants’ ratings who finished the study in under 4 min or over 10 min, as well as those who failed a single attention check.

Both phases of the experiment took roughly 10 min, so participants were paid £1.75 for their time (to be above the National Minimum Wage at the time of the study, and not to penalize participants who were slower/more thorough. Payment was based on the time it takes to complete the study, ensuring all participants are above the National Minimum Wage, leaving plenty of room for error).

### 2.2 ITT passing criteria

Both ITT passing criteria were preregistered before data collection.

1. Absolute Criteria:
  Our absolute passing criteria was if an opponent's argument was rated as either “Agree” or “Strongly Agree” by proponents of that position. That argument was given a “1” if it was given a rating of Agree or Strongly Agree, and zero for all other positions on the scale. Using only Agree and Strongly Agree ensures that we are capturing arguments that proponents are certain that they agree with.
2. Relative Criteria:
  Our relative criteria relies on the distribution of ratings by the raters. We calculated the mean ratings of all proponent arguments, rated by proponents, and used this as the “baseline” measurement for passing the ITT. The mean was rounded to the nearest Likert scale point, and this was then used as the threshold for passing the ITT. Thus, opponent arguments rated by proponents were compared to the baseline threshold; if they were the same or higher, they were given a 1, if they were below, they were given a 0.

### 2.3 Hypotheses

Our first two hypotheses are assumption checks to ensure that there is higher internal agreement within positions than between, that is, that proponents of a position agree with each other's arguments more than opponents do.

#### 2.3.1 Assumption checks

1. Arguments written by proponents, in support of their own position, have higher agreement from proponents than from opponents (argument‐rater incongruence).
2. Arguments written by opponents, in opposition to their own position, have lower agreement from proponents than reasons written by proponents. (arguer‐argument incongruence).

#### 2.3.2 Main hypotheses

1. Those who have spent more time actively learning about the topic are more likely to pass the ITT (e.g., reading articles, blogposts, watching documentaries, or listening to podcasts).
2. Those who have spent more time discussing the topic with those who disagree with them are more likely to pass the ITT.
3. Those who pass the ITT are less likely to think their ideological opponents (a) have bad reasons, (b) are unintelligent/irrational/ignorant, (c) are unethical/immoral/of weak moral character.

### 2.4 Analysis plan

We used pilot data on the topic of vaccination to design our analysis. We collected arguments about vaccination as part of a previous study (Brand & Stafford, 2022), but only used 10 participants’ arguments (out of 700) for pilot analyses.

### 2.5 Statistical models

We ran Bayesian multi‐level binomial logistic regressions with passing the ITT as the binary outcome variable and arguer position as the predictor variable, with varying intercepts for rater ID, arguer ID, and argument ID. Full details of the analysis, which was preregistered, can be found in the repository.

We ran the same models with the discuss and research variables as predictors. In addition to these analyses, we ran ordinal logistic models with the Stanley et al. (2020) measures of open‐mindedness as the outcome variable, and “ITT Passed” as a predictor variable, with arguer ID as a varying intercept, as well as Stanley item as a varying intercept (those that were measuring “good reasons,” “moral character,” and “intelligence” were clustered together). Some measures were reverse‐coded so that higher ratings consistently represent more open‐minded responses. We recode the discuss and research variables as 0/1 depending on if they are above “4” on the 1–7 Likert scale (which means they have reported that they discuss/research the topic either most months, most weeks, or most days, as opposed to a few times a year/once or twice a year/rarely, and never).

We use 89% credible intervals to infer if a parameter has a strong effect on the outcome. For example, if the 89% credible interval crosses zero, we will infer that the parameter did not have a strong effect on the outcome variable. Eighty‐nine percent credible intervals are the default in the Rethinking package (McElreath, 2020) because they discourage people from interpreting them as 95% confidence intervals or in a null‐hypothesis‐testing framework.

### 2.6 Planned exploratory analyses

We explored the Relative ITT criteria alongside the Absolute ITT criteria, as mentioned above. At the point of preregistration, we did not have specific predictions about how they might differ.

We also explored within‐topic differences. It is plausible that minority groups (e.g., vegans and vaccine refusers in our sample) may be better placed to pass the ITT, as they are more likely to be exposed to the opposing view than those in the majority group. The Brexit topic is notable for being both highly politicized and evenly split in the UK voting population (at and after the time of the 2016 referendum). The vaccination topic is equally politicized, since the coronavirus pandemic started in 2020, but with a majority (pro‐vaccination) and minority (anti‐vaccination) positions. Comparisons across topics were preregistered with no specific directional predictions.

## 3 Results

### 3.1 Assumption checks

Our two preliminary hypotheses were assumption checks. First, we confirmed that arguments written by proponents have higher agreement from proponents than opponents. This was true in all cases except for non‐vegans; whereas vegans rated pro‐vegan arguments on average as 6, and anti‐vegan arguments as 2.27, non‐vegans were ambivalent toward both arguments for and against veganism, rating pro‐vegan arguments as 4.55 on average and anti‐vegan arguments as 4.49 on average—thus agreeing with pro‐vegan arguments slightly more than anti‐vegan arguments. Second, we confirmed that opponent arguments have lower agreement from proponents than the reasons written by proponents. This was true in all cases (see Supplementary Material for all rating combinations broken down by arguer position, argument position, and rater position).

### 3.2 Main hypotheses

In support of our preregistered predictions, passing the ITT predicted participants’ responses on the Stanley measure of open‐mindedness (e.g., whether you think your ideological opponents have good reasons for their position), in those topics for which we had Stanley measures: (Brexit mean estimate: 0.08, 89% CI: [0.01, 0.16], Veganism mean estimate: 1.14, 89% CI: [1.07, 1.22]). Passing the ITT also predicted participants’ responses to whether they think their ideological opponents are immoral (Brexit: −0.16 [−0.20, −0.11], Veganism: −1.12 [−1.16, −1.07]) and ignorant: (Brexit: −0.20 [−0.24, −0.16], Veganism −0.56: [−0.60, −0.51]), in that they were less likely to think their ideological opponents were ignorant or immoral, if they passed the ITT. On the cumulative probability scale, this represents a change from 38% (failed ITT) to 41% (passed ITT) of participants either disagreeing or strongly disagreeing that their opponents are ignorant of the Brexit topic. In the veganism topic, it represents a change from 44% (failed ITT) to 70% (passed ITT) of participants either disagreeing or strongly disagreeing that their opponents are immoral. (Note this change is ameliorated from 56% [fail] to 58% [pass] when using the relative passing criteria, as veganism showed a difference in relative and absolute passing rates, shown in Table 1). Full tables of the predicted probabilities of Likert responses of the open‐mindedness measures for passing and failing the ITT test are available in the repository, as are plots of the predicted changes in absolute probability on the Likert‐scale measures for passing and failing the ITT.

**Table 1:** Mean (and standard deviation) ratings of the Baseline and ITT arguments, per Arguer position

ARGUER	Mean Baseline rating (Proposing arguments, rated by Proponents)	Mean ITT rating (Opposing arguments, rated by Opponents)
**Vegan**	6.00 [1.32]	4.46 [1.81]
**Non‐vegan**	4.49 [1.80]	5.81 [1.60]
**For Vaccines**	5.96 [1.28]	5.00 [1.96]
**Against Vaccines**	5.57 [1.72]	5.21 [1.71]
**Leaver (For Brexit)**	5.23 [1.45]	4.88 [1.84]
**Remainer (Against Brexit)**	5.12 [1.73]	4.97 [1.74]

*Note*. Baseline arguments are Arguer‐Argument‐Rater congruent (thus proposing arguments rated by proponents). ITT arguments are Arguer‐Argument incongruent and rated by opponents (thus opposing arguments rated by opponents).

Against our preregistered predictions, we found no evidence that passing ITT is predicted by self‐reported time spent actively researching the topic (Brexit: [−0.50, 0.01], Covid‐19 Vaccines: 89% CI: [−0.21, 0.39], Veganism: [−0.42, 0.09]), nor discussing the topic with those that disagree with you, (Brexit: [−0.75, 0.13], Covid‐19 Vaccines: [−0.62, −0.03], Veganism: [−0.39, 0.25]), although this was not the case for the absolute passing criteria for veganism, in which researching and discussing more often both meant participants were less likely to pass the absolute ITT criteria (Researching: [−0.87, −0.36], Discussing: [−0.74, −0.12]), opposite to our prediction.

### 3.3 Planned exploratory analysis

**Fig. 1:**
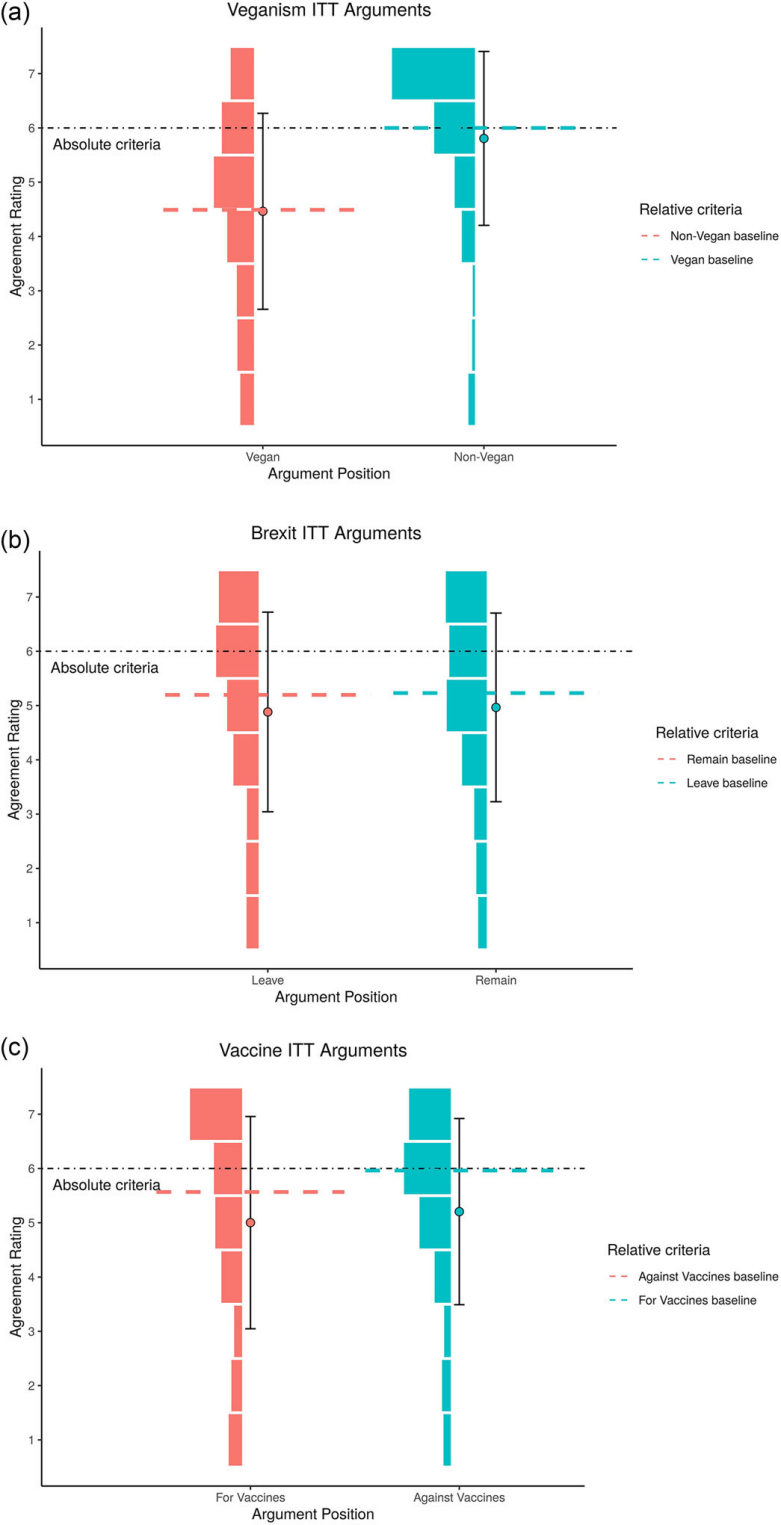
Distribution of ITT argument ratings, compared to the relative and absolute passing criteria for topics: (a) veganism, (b) brexit, and (c) vaccination. The relative passing criteria is the mean rating of the baseline arguments for the opposing position that participants are masquerading as. 1 = Strongly Disagree, 7 = Strongly Agree. It is important to note, raters did not know either the argument's or the arguer's position when asked to rate their agreement with the arguments.

We found no evidence that one position was more likely to pass the ITT than another, when controlling for variation in rater, arguer, and argument, against our exploratory prediction, except for non‐vegans in the absolute criteria, in that non‐vegans were more likely to pass the absolute criteria: Vegan Absolute criteria: 89% CI: [−2.84, −2.08], relative criteria: [−0.37, 0.35], Brexit Absolute criteria: [−0.33 0.55], Brexit Relative criteria: [−0.51, 0.21], Vaccination Absolute criteria: [−0.76, 0.04], Vaccination Relative criteria: [−0.76, 0.05]. ITT argument ratings are shown in Fig. 1. The mean baseline and ITT ratings per topic are shown in Table 1. The proportion of arguments passing and failing the ITT test is shown in Table 2.

**Table 2:** Proportion of arguments successfully passing both the Absolute and Relative Ideological Turing Test criteria, by topic and position

	ITT Criteria	
Arguer Position	Absolute	Relative
Vegans (For veganism)	0.32	0.71
Non‐vegans (Against veganism)	0.71	0.71
Against Vaccination	0.54	0.54
For Vaccination	0.49	0.54
Leaver (For Brexit)	0.46	0.64
Remainer (Against Brexit)	0.44	0.66

## 4 Discussion

In this paper, we report an operationalization of the ITT, to our knowledge, for the first time. The measure asks participants to provide arguments for the opposite side of their own view, with those arguments then rated by proponents of that view. An argument has “passed” the ITT if it is rated as highly, if not higher, than the average arguments provided by proponents of that view, rated by those proponents. This “relative” criterion takes into account the agreement within proponents of the same argument, as a baseline to compare opponent ratings. We developed this measure with three separate, often polarizing topics: Brexit, Covid‐19 vaccinations, and veganism. We found that, when asked to give three reasons for and against their position, participants unsurprisingly agree with reasons provided by their own ideological proponents far more than those provided by their ideological *opponents*. On the whole, participants from both sides, across all topics, had comparable performance in at providing arguments which passed the relative criteria; however, there was variation in the pass‐rate between topics. Only around 54% arguments passed within the topic of Covid‐19 vaccinations, whereas around 71% passed in the topic of veganism, with Brexit achieving around a 64% pass rate for both sides. When accounting for variation within and between arguers, raters, and arguments, we found no evidence that either side was more likely to “pass” the test within each topic. It remains open to interpretation whether these figures represent a surprising failure of individuals to provide arguments that are accepted by their co‐proponents, or a surprising success of individuals in being able to articulate arguments that are accepted by their opponents.

Between‐topic differences also demonstrate the importance of collecting baseline ratings, to allow investigation of relative as well as absolute criteria (i.e., just achieving a rating of either agree or strongly agree). For example, non‐vegans appear to outperform vegans in their ITT attempts when looking at the absolute criteria; however, when looking at the relative criteria, it reveals that this result is an artifact of vegans showing a much higher tendency to agree with all pro‐vegan arguments in general, and non‐vegans showing ambivalence toward both pro‐ and anti‐vegan arguments, choosing “neither agree nor disagree” predominantly for both argument types. Asking participants to provide proponent as well as opponent arguments, and asking raters to rate both positions, provides insights into variation in agreement within and between topics and positions. This is important for the potential future implementation of this task. It would be possible to collect a briefer version of the task by only asking participants for opponent reasons (ITT arguments), and only getting proponent raters to rate those ITT attempts. However, without the baseline measures of proponent's ratings of their fellow proponent's arguments, and vice versa, there would be a risk of missing these nuances and granting one side a false failure/success rate based on the “absolute criteria” alone, rather than taking into account the “relative criteria” as we have.

In line with our preregistered prediction, those who could pass the ITT were more likely to rate their ideological opponents as having “good reasons” for their position, were less likely to rate their opponents as ignorant, unintelligent, or irrational, and were less likely to rate their opponents as immoral, of bad moral character, or unethical. This result replicates the results of Stanley and colleagues, who found that exposure to opposing reasons reduces negative impressions of ideological opponents (Stanley et al., 2020). To the extent this holds, this association would suggest our measure goes hand‐in‐hand with being nonjudgmental and truly “open‐minded” toward your ideological opponents, and so reflects some validation of these self‐report measures. The positive association could come about in multiple ways; either someone is inherently less judgmental of their ideological opponents, and is thus more charitable or effortful in their attempts to create good reasons for the other side's perspective—or—*because* someone is better able to represent their opponent's views, they understand that their opponents have good reasons and *thus* do not judge them to be ignorant, immoral, and so on. Similarly, those who have either been exposed to, learnt about, or are better integrated with their ideological opponents and hence their reasons, might not only think more highly of their opponents but would also be able to better characterize their position because of this exposure/integration.

The above explanation is not supported by our finding that those who report spending more time either researching/reading about the topic, or discussing the topic with those who disagree with them, are *not* more likely to pass the ITT. One explanation for this latter finding could be that those who spend a lot of time researching/discussing their position may become even more polarized/isolated from their opponents, depending on the nature of their exposure. For example, within the veganism topic, those who report spending a lot of time researching the topic/discussing it with others were even less likely to pass the ITT according to the absolute criteria (i.e., the ability to produce arguments rated as “Somewhat Agree” or “Agree” by alternative view holders). This result may have been confounded by the fact that vegans are of course more likely to discuss veganism than non‐vegans, or may have researched the topic to such an extent that they have become unable to relate to/empathize with reasons for the opposite position. Other explanations could be that either our self‐report measure of time spent researching or discussing a topic is error prone, or that the amount of time reported does not reflect the quality or diversity of people or arguments that the person is exposed to.

One thing that is clear from our research is that we should not expect open‐mindedness or perspective‐taking to be the same for all topics, and we should question whether a “trait” of open‐mindedness that is consistent within individuals across topics, context, and time is realistic. Given that we know that human beliefs, emotions, norms, and behaviors are all shaped heavily by social learning and interaction with other people, we should expect open‐mindedness to vary according to individuals’ exposure to different perspectives, different arguments, different people, and different contexts. In this way, it is reasonable to believe that open‐mindedness should be a state—based on your particular experience of that domain—not a life‐long trait. If open‐mindedness is fundamentally about not thinking ill of those who hold a view different to your own, acknowledging that they have their own evidence, be that experiential or empirical, to support their belief, then this “ability” should change depending on exposure to those individuals. This line of reasoning is supported by evidence that contact and integration with migrant communities reduces negative stereotypes of immigrants (Matejskova & Leitner 2011; Pettigrew & Tropp 2006; Zhou, Page‐Gould, Aron, Moyer, & Hewstone, 2019), as well as evidence that consultation with stakeholders and communities leads to better policy‐making decisions (Beierle 2002; Irvin & Stansbury 2004). Of course, it is conceivable that if you experience your own perspective changing on multiple topics over time, or you are exposed to a diverse variety of opinions and beliefs about many topics over time, those experiences may lead to a tendency (or “trait”) of being more “open” to new opinions and evidence in general.

A fundamental aspect of the ITT is the ability to represent a reason from the population that you disagree with, but specifically a reason that a random subsample from that population would agree with. In this respect, it is important to be familiar with the total pool of reasons, particularly those that are most commonly offered by that population (and hence more likely to be agreed with by a sample of that population). For this reason, our test purposefully does not require participants to generate good, logical, or coherent reasons. We specifically worded our ITT question to be “what would they say?” and the rating wording to be “how much do you agree?”, and purposely not “what is the best reason?” or “how good is this reason?” This is because we assume there is a pool of reasons for any particular belief that vary in their logical coherence and evidence quality, but that the true value of open‐mindedness comes from having an appreciation and awareness of this variety of reasons, rather than being able to construct a single “best” reason in terms of evidence or logic.

The ability to represent others’ reasons connects to a rich body of research in cognitive science on perspective‐taking and theory of mind (Nichols & Stich, 2003). In particular, there may be analogues of our measure in so‐called “advanced theory of mind” tests (Happé, 1994; Osterhaus & Bosacki, 2022), which typically use vignettes to assess participant's perspective‐taking and sensitivity to subtle social cues. We suspect that individual differences in theory of mind and related capacities for perspective‐taking and mimicry impact the ability to articulate reasons for positions you disagree with, as captured by the ITT. While we are unable to confirm this with the current data, investigation of the relation may be a productive avenue for future research.

In this paper, we operationalize a behavioral measure of open‐mindedness, namely, the ITT. We found that the ability to “pass” this test predicts open‐mindedness toward ideological opponents; in that those who pass the test are less likely to think their opponents are ignorant, unintelligent, irrational, or immoral. Participants who pass are also more likely to think their ideological opponents have good reasons for their position. We did not find support for the prediction that those who self‐report spending more time discussing the topic with those who disagree with them are better able to pass the ITT, nor did we find that those who self‐report spending more time researching the topic are better able to pass the ITT test.

We found significant variation between topics in the ability to pass the test, but we did not find that the minority position, or either “side” of a debate were more or less likely to pass the test. Contrary to the assumption that disagreement across lines of polarization is associated with ignorance of opponents, we found evidence that participants across both sides of our three topics were competent at producing arguments which would be endorsed by their opponents. Aside from the substantive empirical results, our primary contribution is methodological. Open‐mindedness has hitherto been assessed by self‐report measures. We take a concept with prima facie validity—the ITT—and show that it can be operationalized in a behavioral measure. We suggest that this behavioral task may be a useful tool for testing the success of interventions in depolarization and perspective‐taking.
